# Optimizing diffusion of an online computer tailored lifestyle program: a study protocol

**DOI:** 10.1186/1471-2458-11-480

**Published:** 2011-06-20

**Authors:** Francine Schneider, Liesbeth ADM van Osch, Stef PJ Kremers, Daniela N Schulz, Mathieu JG van Adrichem, Hein de Vries

**Affiliations:** 1 CAPHRI/Department of Health Promotion, Maastricht University, P.O. Box 616, 6200 MD Maastricht, the Netherlands; 2 Nutrition and Toxicology Research Institute Maastricht (NUTRIM)/Department of Health Promotion, Maastricht University, P.O. Box 616, 6200 MD Maastricht, the Netherlands

## Abstract

**Background:**

Although the Internet is a promising medium to offer lifestyle interventions to large amounts of people at relatively low costs and effort, actual exposure rates of these interventions fail to meet the high expectations. Since public health impact of interventions is determined by intervention efficacy and level of exposure to the intervention, it is imperative to put effort in optimal dissemination. The present project attempts to optimize the dissemination process of a new online computer tailored generic lifestyle program by carefully studying the adoption process and developing a strategy to achieve sustained use of the program.

**Methods/Design:**

A prospective study will be conducted to yield relevant information concerning the adoption process by studying the level of adoption of the program, determinants involved in adoption and characteristics of adopters and non-adopters as well as satisfied and unsatisfied users. Furthermore, a randomized control trial will be conducted to the test the effectiveness of a proactive strategy using periodic e-mail prompts in optimizing sustained use of the new program.

**Discussion:**

Closely mapping the adoption process will gain insight in characteristics of adopters and non-adopters and satisfied and unsatisfied users. This insight can be used to further optimize the program by making it more suitable for a wider range of users, or to develop adjusted interventions to attract subgroups of users that are not reached or satisfied with the initial intervention. Furthermore, by studying the effect of a proactive strategy using period prompts compared to a reactive strategy to stimulate sustained use of the intervention and, possibly, behaviour change, specific recommendations on the use and the application of prompts in online lifestyle interventions can be developed.

**Trial registration:**

Dutch Trial Register NTR1786 and Medical Ethics Committee of Maastricht University and the University Hospital Maastricht (NL2723506809/MEC0903016).

## Background

Due to its high accessibility and its potential to reach large numbers of people, the Internet is considered to be a promising medium in the field of health promotion for offering tailored and targeted behaviour change programs to the general public [1-5]. Over the recent years, positive effects of online interventions applying computer tailored (CT) techniques have been reported [2,6,7], addressing physical activity [8,9], fruit and vegetable intake [10,11], smoking [12-15] and alcohol consumption [16,17]. However, in sharp contrast to these high promises, actual reach of these interventions is failing to live up to the high expectations [18-23].

Since public health impact of interventions is determined by their efficacy and exposure in the target group [24,25], it is important to optimize exposure to these interventions by conducting implementation studies and optimizing the methods by which they are disseminated to the general public. Earlier studies defined exposure as pertaining to three different aspects; accessing the intervention (first use), staying on the intervention for a substantial period of time (prolonged use) and revisiting the intervention (sustained use) [19]. Adoption, or first use, rates of online interventions are generally considered to be low [19,25,26], causing actual levels of engagement in the intervention as well as adherence to the intervention to be even lower. Since health behaviour change is a complex process, achieving sustained behaviour change is not only dependent on adoption of a behaviour change program. During a visit, intensive engagement in the program is essential to allow further notification of its content and involvement in its effective components [20,27]. Furthermore, the number of visits to an intervention is important. Due to a high dose-response relation [28,29], sustained use of the program is essential to further maximize its effect on subsequent health behaviour change [30,31].

The current project aims at optimizing exposure rates of a combination of several effective online CT programs, targeting physical activity, fruit and vegetable consumption, smoking and alcohol consumption, offered through the Internet. This combined CT program will be integrated in the Dutch Adult Health Monitor, a monitoring tool used by all Regional Public Health Service (RPHS) in the Netherlands in order to assess different aspects of general health and health related topics at fixed points in time. This integration will result in an online program that respondents of the Dutch Adult Health Monitor can turn to in order to obtain detailed and personalized information regarding their current health behaviour status, as well as personalized advice on how to improve their health by focusing on the key health behaviours.

Several theories underline the importance of different processes to foster effective diffusion of interventions [26,32]. These processes pertain to actual adoption (or first use) of the intervention by the target group and development of strategies to achieve sustained use of the intervention over time. In order to increase adoption rates of an intervention, it is imperative to obtain detailed profiles of those who successfully adopt a new intervention, i.e. so-called innovators or early adopters [25,26]. It is important to reach those people that benefit most from online lifestyle interventions, i.e. the people that engage in risk behaviours like smoking, excessive alcohol use, lack of physical activity or unhealthy eating patterns. By studying characteristics of first-time users and mapping the way the program is used and re-used, detailed knowledge on who adopts the intervention and on determinants of adoption can be obtained. Subsequently, this knowledge can be used to further optimize intervention content, as well as dissemination strategies in order to reach the target group. Furthermore, if first use is established, effective strategies need to be developed and tested in order to attain sustained use by first time users.

Currently computer tailored interventions are offered reactively to the public, implying a passive approach in which users themselves must undertake action in order to benefit from the intervention [33]. Prior research has, however, demonstrated that attaining visitors' loyalty to an intervention over an extended period of time is a very effortful process, entailing continuous endeavours to keep users satisfied and interested [20,22,29]. The suboptimal levels of exposure that are currently reached with the use of reactive strategies, combined with the high levels of attrition, indicate that the efforts put into ensuring visitor's loyalty are inadequate [27]. Therefore, focus should be directed towards developing and testing more proactive approaches in order to increase sustained use of effective behaviour change programs. Results from a recent review demonstrate the advantages of using periodic prompts as a relatively cost-effective method to encourage involvement in health promoting interventions [34]. However, since most studies included in this review refrained from using control groups and long-term data collection, to date, effectiveness of periodic prompting cannot be firmly established. Additional research to reliably assess the effectiveness of proactive strategies, such as prompting, is therefore needed.

In order to optimally disseminate the program, the present research project will thoroughly investigate adoption of an online CT lifestyle program and development of strategies to achieve sustained use. First, studying the adoption process will yield results on the level of adoption, determinants involved in adoption and characteristics of adopters and non-adopters as well as satisfied and unsatisfied users. A strategy aimed at increased and sustained level of use will be designed and tested. On that account the efficacy of a *pro-active approach *using periodic e-mail prompts in order to obtain sustained use of the intervention over time will be studied in a randomized control trial.

## Methods/design

The current project emphasizes the processes of first use (adoption) and re-use of a combined computer tailored online lifestyle program, by examining each process in a single study. First, a general description of the CT program will be provided, before proceeding to the detailed description of the separate studies.

The current project is approved by the Medical Ethics Committee of Maastricht University and the University Hospital Maastricht (NL27235.068.09/MEC 09-03-016), and is registered with the Dutch Trial Register (NTR1768).

### The CT program

The new CT program integrates established CT programs tested and proven to be effective in randomized control trials for increasing smoking cessation, promoting the intake of fruit and vegetables, increasing the level of physical activity and reducing the consumption of alcohol [16,35-38]. The multi-component CT program is embedded in an already existing channel; the Adult Health Monitor used by all Dutch RPHS's. This combination results in an online CT program that a substantial proportion of the Dutch population can use in order to obtain detailed and personalized information regarding the status of their current health behaviours, as well as personalized advice on how to positively change their health by focusing on key determinants of the five health behaviours.

#### The Adult Health Monitor

The Adult Health Monitor takes place with an interval of four years and serves as a monitoring tool used by the RPHS's to assess the overall level of health in the Dutch population by approaching a representative sample of the population to fill out a questionnaire assessing different aspects of general health (e.g. physical and mental health) and health related topics (e.g. social and physical environment) [39]. Physical health entails the five key behaviours targeted in this intervention; *physical activity*, measured by the Short QUestionnaire to ASsess Health enhancing physical activity (SQUASH) [40], *fruit and vegetable intake*, measured by the Short Questionnaire for Fruit and Vegetable Intake [41,42], *alcohol consumption*, measured by the Dutch Quantity-Frequency-Variability Questionnaire (QFV) [43] and *smoking*, measured by the abbreviated version of the Fagerström Test for Nicotine Dependence [44]. Results obtained through this Adult Health Monitor are communicated to the concerning municipalities, which subsequently use these as a basis for future health policies.

Integrating the new CT program into the Adult Health Monitor may have several benefits. Firstly, it can enable the RPHS's to optimize their public health education task; besides solely monitoring health behaviour, the RPHS's now have an opportunity to provide people with personalized advice on how to change their health behaviours. Furthermore, it may provide the random sample of adults included in the Adult Health Monitor an incentive for their participation and might serve as a cue to action for them to positively change their health behaviour. Since the current CT program is embedded in the Adult Health Monitor of the Provinces of Limburg, Brabant and Zeeland, with an approximate total reach of 100.000 inhabitants, this integration also provides an important access point for reaching a considerably large segment of the Dutch population with a lifestyle intervention.

#### Content of the CT program

The CT program uses a step-by-step approach to guide people towards behaviour change. After online completion of the Adult Health Monitor people are informed in detail on the embedded program and the accompanied studies. People that indicate to be interested in the program are subsequently linked to the program. During their first visit demographic data (age, gender, height, weight, religious background, ethnicity, educational level and income) and personal data concerning the target health behaviours, acquired through the Adult Health Monitor questionnaire, are transferred to the CT program and used to inform people on the current status of their health behaviours by comparing these to Dutch public health guidelines (i.e. being moderately physically active for 30 minutes at least 5 days a week, eating 2 pieces of fruit per day, eating 200 grams of vegetables per day, not drinking more than one (women) or two (men) glasses of alcohol a day and non-smoking). In case of discrepancies, people are alerted and provided assistance in changing their health behaviour(s) with help of CT advices available per behaviour.

Changing health behaviours with help of computer tailoring is the main component of the program. A CT module is available per health behaviour and within each module people have the opportunity to obtain personalized advices regarding the opted health behaviour. All health behavioural advices are adapted to individuals' characteristics by considering demographic, behavioural and social-cognitive variables [24,45,46]. Some variables, such as individuals' current health behaviours and characteristics of the respondent (gender), are directly obtained through the Adult Health Monitor. Other variables, such as cognitive variables with regard to the health behaviour (attitude, perceived social influence and self-efficacy), intention to change the health behaviour and planning strategies (action plans and coping plans) are assessed by using an additional tailoring questionnaire. All tailoring components are based on the Integrated Model for exploring motivational and behavioural Change (I-Change Model) [47].

### Intervention materials

Message content and algorithms of the CT advices are based on prior theory and evidence based CT interventions on physical activity, fruit and vegetable intake, alcohol consumption and smoking cessation [16,35,37,38,48]. Online questionnaires available for the health behaviours serve as basis for the CT advices. Attitude is measured by investigating respondent's level of agreement to a total of 6 statements regarding the pros (3 statements) and cons (3 statements) of the health behaviour under consideration (five-point scale; e.g. 'When I quit smoking, my health will improve', 'When I quit smoking, I will gain weight'). Social influence is measured by three different concepts; social norms, social modelling and social support. Social norms are assessed with one item, asking respondents to complete statements regarding perceived support from people in their environment (i.e., partner, family members, friends and colleagues) regarding the health behaviour (five-point scale; e.g. 'According to the people within my direct environment.... I certainly should not smoke'). Social modelling is assessed by one item asking participants how many people within their environment (i.e., partner, family members, friends and colleagues) engage in the health behaviour under consideration (five point scale; e.g. 'How many of your family members eat at least two pieces of fruit every day?'). Social support is measured with one item assessing the degree of support respondents receive from the people within their direct environment (i.e., partner, family members, friends and colleagues) to engage in the health behaviour (four-point scale; e.g. 'Do your friends stimulate you to be physically active?'). Self-efficacy is measured by means of 6 items asking participants to indicate whether they would be able to engage in (physical activity and nutrition) or refrain from (smoking and alcohol consumption) the key health behaviour when encountering difficult situations (seven-point scale; e.g. 'Will you be able to eat a sufficient daily amount of vegetables when other tempting alternatives are within reach?'). The intention to change behaviour is measured by means of two items. The first item is an extended version of the Stages of Change concept [49,50] using an algorithm consisting of ten stages varying from unaware to action, whereas the second item assesses the degree to which people are considering to engage in the key behaviour in the future; (seven-point scale; e.g. 'Do you intend to quit smoking in the future?'). Action planning is measured by three items asking respondents to indicate whether they plan to use a particular preparatory strategy when engaging in a certain behaviour (five-point scale; e.g. 'Are you planning to join a sports club?'). Coping planning is assessed by six questions asking participants to what degree they have made a specific plan regarding how to cope with difficult situations that might hinder execution of the health behaviour. These questions are based on the difficult situations used for the self-efficacy items (five-point scale; e.g. 'I have made a detailed plan to refrain from smoking when I just finished a meal.').

### Study 1: Assessing the adoption process of the CT program

The main goal of the first study is to assess the level of first time use of the new CT program, frequency of use, level of satisfaction with the new CT program by participants of the Adult Health Monitor 2009 and determinants of adoption and use. Furthermore, characteristics of users and non-users and satisfied and unsatisfied users will be investigated.

### Design

This is a prospective study, employing a longitudinal design. At baseline participants of the Adult Health Monitor interested in the new CT program receive feedback concerning their current health behaviour. In case of a negative discrepancy between the status of their current health behaviours and public health guidelines set for these behaviours participants are stimulated to change these behaviours with help of the CT program. During a period of three months, use of the CT program is monitored anonymously resulting in a detailed overview of number of first visits, revisits, and the specific health behaviours selected.

### Recruitment and procedure

The research sample consists of Dutch adults participating in the Adult Health Monitor 2009 of the Dutch Provinces of Limburg, Brabant and Zeeland. The RPHS's will invite a representative sample of inhabitants of the provinces North-Brabant, Zeeland and Limburg (+/- 100.000) by mail to participate in the Monitor. Participants will be offered the opportunity to fill out a written questionnaire, which is included, or to complete an online version of the Monitor questionnaire. Electronic completion will be highly encouraged since the new CT program is an online program only accessible for respondents participating via the Internet. Based on previous experiences with this Monitor it is expected that a response rate of approximately 33% will be reached, indicating that about 33.000 respondents will fill out the Adult Health Monitor.

**Figure 1:**
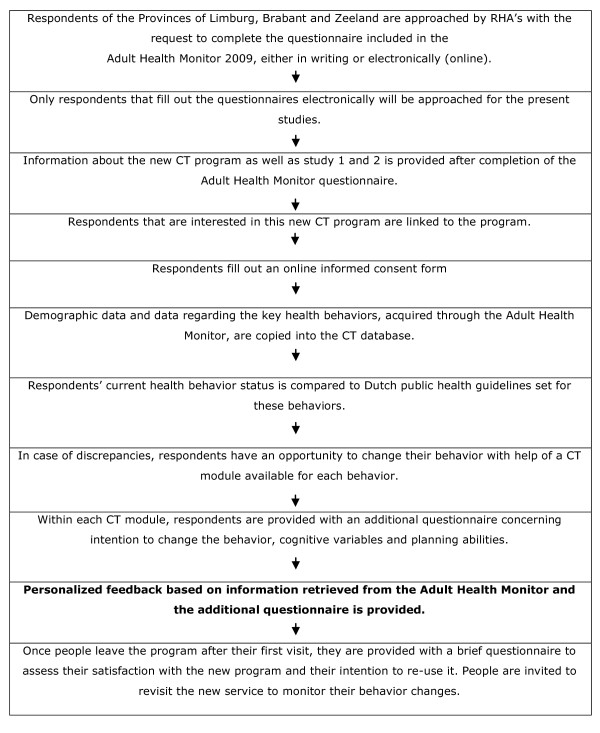
**Flowchart study 1**.

After completing the Adult Health Monitor, all participants of the *online *version are introduced to the new embedded program, which is free of charge and consists of an opportunity to receive CT feedback about their health behaviour. Participants indicating to be interested in the new program will be asked to leave their e-mail address and receive an e-mail including an invitation to log in to the CT program with a personal login code and password approximately three weeks after completion of the Monitor. After logging on to the program participants receive detailed information on the goal and the content of both studies described in this protocol. After receiving this information, participants are asked to fill out an online informed consent form. Only those participants that consent to participation are directed to the new service. Subsequently, data on demographics and the five health behaviours obtained through the Monitor are transported to the new program, resulting in a personal overview in which an individual's current health behaviour status is compared to the guidelines set for these health behaviours. Within the program, five modules generating CT health advice are available to assist participants in changing their current health behaviour status when needed. Once respondents decide to leave the CT program after their first visit, they will be asked to fill out an additional questionnaire to assess their satisfaction with the program and their intention to revisit the program again in the near future (Figure 1).

### Inclusion and exclusion criteria for participants

Participants can be included in the current study when they are between 18 and 65 years and able to understand the Dutch language sufficiently. Moreover, they have to have access to the Internet. There are no explicit exclusion criteria stated for the current study.

### Measures

#### User characteristics

User characteristics are collected to produce a detailed user profile entailing information on personal characteristics, health behaviour status as well as socio-cognitive characteristics. Questions used in the Health Monitor pertaining to personal characteristics, included questions on gender, height, weight, marital status, religious background, ethnicity, educational level, current work status, and income. Furthermore, questions used in the Health Monitor pertaining to physical activity [40], fruit and vegetable intake [41], alcohol intake [43] and smoking behaviour [44] were used to assess health behaviour status. Finally, several determinants of health behaviour as mentioned in the I-Change model were used as socio-cognitive characteristics; intention, attitude, social influence, self-efficacy, action and coping planning.

#### Program evaluation

Satisfaction with the CT program will be assessed by using a total of five items; four items measuring the degree to which respondents regard the information provided within the program as new, personal, useful and encouraging (five-point scale; e.g. 'I think the information within the CT program is useful') and one item assessing overall satisfaction (ten-point scale; e.g. 'How satisfied are you with the current program?').

#### Intention to re-use the CT program

Intention to re-use the CT program is assessed by using a 10 point intention scale ('I intend to use this CT program again in the near future').

### Outcome measures

To measure the adoption level of the new CT program focus will be on level of first use, demographic and behavioural characteristics of users and non-users, satisfaction with the program, characteristics of satisfied and unsatisfied users, intention to re-visit the program and frequency of re-visits. In order to measure level of first use of the program, two dichotomous variables will be created; one indicating whether participants initiate a behaviour change module and one indicating whether participants finish the module. Initiating a module is labelled as "yes" when people fill out the first question of this module, which is the question concerning intention to change the specific health behaviour. Completion of a module is labelled as "yes" when people also fill out the final question of the module. Re-visiting of the program will be assessed monitoring whether participants log in to the program within a three month period after baseline (0 = yes/1 = no). Re-visits to the program will be measured by comparing the dates of log-in to the baseline date.

### Statistical analysis

First, general descriptive statistics will be used to describe personal characteristics of the participants, as well as main findings concerning current health behaviour status, adherence to the public health guidelines and socio-cognitive characteristics. Second, logistic regression analyses will be conducted with initiation of a module, completion of a module and revisiting of the program as dependent variables. Demographics, such as gender, height, weight, marital status, religious background, ethnicity, educational level, current work status, income, health behaviour status and cognitions (measured at baseline) are included in the model as predictors.

### Study 2. Increasing sustained use of the CT program

The aim of this study is to test the effect of using a proactive approach (i.e. periodic e-mail prompting) on revisiting of the program compared to a reactive approach. Furthermore, the efficacy of these approaches with regard to behaviour change will be studied.

### Design

The second study consists of a randomized control trial (RCT) including one experimental group. A design with a baseline measurement and three follow-up measurements (6, 12 and 18 months) measuring health behaviour status, intention to change, cognitive variables and planning abilities, will be used to compare the experimental condition (use of a proactive technique) to a control condition (use of a reactive approach).

### Recruitment and procedure

**Figure 2:**
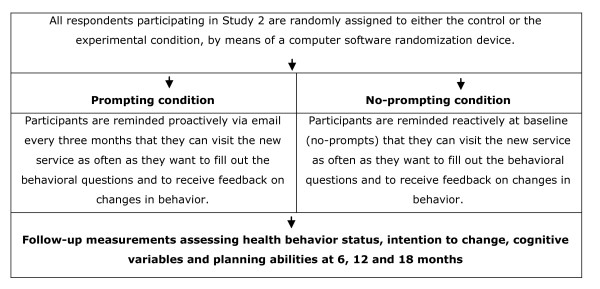
**Flowchart study 2**.

The research sample consists of individuals that participate in the Dutch Adult Health Monitor and that decide to pay a first visit to the new CT program. After logging in to the program at baseline and consenting to participation by filling out an online informed consent form, participants are randomly assigned to one of the two conditions. People in the experimental condition are reminded proactively via e-mail every three months for a period of 18 months to visit the CT program in order to obtain iterative feedback concerning their health behaviour status and additional CT advice, whereas people in the control condition do not receive any additional prompts and are only encouraged to re-visit the program in the future at baseline. All participants will be invited by e-mail for the follow-up measurements at 6, 12 and 18 months (Figure 2.).

### Inclusion and exclusion criteria for participants

Participants can be included in the current study when they are between 18 and 65 years and able to understand the Dutch language sufficiently. Moreover, they have to have access to the Internet. There are no explicit exclusion criteria stated for the current study.

### Materials

Three months after their first visit, participants in the experimental condition receive an e-mail, prompting them to revisit the program. This e-mail opens with a personal salutation and reminds people about their first visit to the program. Also, in short, the content and purpose of the program will be summarized, in case people have forgotten about the details of the program. Subsequently, people are invited to revisit the program in order to obtain information on their current health status and to monitor their progress. Participants were also pointed out the opportunity to receive additional, iterative, health advice on the health behaviour selected at baseline or on a new behaviour. In order to facilitate logging in to the program, the e-mail will also contain details on personal log-in information (user name and password).

### Randomization

Interested participants are directed through the Adult Health Monitor to the new program, where they can log in to the program with a personal username and password. After signing in to the program, participants will be randomly allocated to either the control condition or the experimental condition. Randomization will occur at the respondent level by means of a computer software randomization device. Blinding of participants is not possible, since participants will take notice of the fact that they receive additional e-mail prompts or not.

### Outcome measures

The primary outcome measures are re-visits to the program and level of behaviour change. Program revisits are objectively monitored for a total period of eighteen months. Recordings will be done anonymously and will yield information about the number of times the program is visited, the duration of each visit (in minutes) and which specific behaviours are most frequently selected. Level of behavioural change is assessed by studying participants' adherence to public health guidelines set for these behaviours (0 = not complying with recommendations/1 = complying with recommendations).

### Secondary outcome measures

Secondary outcome measures include overall tobacco use (number of cigarettes/day), level of physical activity (minutes/day), and overall level of fruit (pieces/day), vegetables (grams/day) and alcohol consumption (glasses/day). Furthermore, socio-cognitive measures, such as intention, attitude, social influence, self efficacy, action and coping planning will be measured.

### Statistical analysis

#### Sample size and power

With regard to re-visits to the program (first primary outcome measure), it is estimated that approximately 50% of the respondents of the no-prompting condition will visit the site more often than four times (after the baseline, and the first three post tests after six, twelve and eighteen months). It is estimated that in the prompting condition 70% of the respondents will visit the site more than four times. Power calculations reveal that in order to be able to detect this difference with a power of 80% (p = .05) at least 186 persons per condition are needed. Taking into account potential attrition, it is proposed to collect the data of 250 persons per cell.

The second primary outcome measure is the level of health behaviour change. The current program entails a total of five health behaviours. Therefore, with regard to the assessment of behaviour change (second outcome measure), estimations for finding effects will be based on smoking, since this behaviour results in the most conservative power calculations. Approximately 25% to 30% of the population smoke [51]. Power estimations are based on the ability to detect a difference in quit rates of 10% with a power of .80 and a *p*-value of .05. It is estimated that, in order to detect this significant difference in quit rates, 219 smoking persons per cell are needed, resulting in a total of 438 smokers [52]. To be able to have 438 smokers in the sample (under the condition that 30% of the Dutch population smokes), 1575 participants need to be recruited. Accounting for 35% attrition during the trial period, the total sample size for this study will be approximately 4500 persons.

#### Analyses

First, general descriptive statistics will be used to describe personal characteristics of the participants, as well as findings concerning current health behaviour status, adherence to the public health guidelines and socio-cognitive characteristics. Second, logistic regression analyses will be conducted with re-visits to the program and level of behaviour change as dependent variables. Furthermore, linear regression analyses will be conducted with number of re-visits, duration of re-visits and number of behaviours selected as dependent variables. Demographics, such as gender, height, weight, marital status, religious background, ethnicity, educational level, current work status, and income, health behaviour status and cognitions (measured at baseline) will be included in the model as predictors of the dependent variables.

## Discussion

Although the Internet is a promising medium to offer lifestyle interventions [5], it is not yet used to its full potential, since actual exposure to online lifestyle interventions is falling short to the high expectations [21,53]. Since public health impact of interventions is determined by efficacy and actual use of the intervention by the target group [19,25], it is necessary to ensure optimal reach of and exposure to these interventions. The current implementation project aims at optimizing the methods by which effective computer tailored programs are disseminated to the general public.

Since optimal dissemination largely depends on the processes of actual *adoption *of the intervention and *development *of a strategy to achieve sustained use of the intervention over time [26], each process is thoroughly and independently studied. Firstly, closely mapping the adoption process of the intervention will gain insight on characteristics of adopters and non-adopters and satisfied and unsatisfied users, as well as determinants of adoption and use. This acquired knowledge can be used to optimize the existing intervention by making it more suitable for the intended target group of Dutch adults aged 18 to 65 years, or to develop adjusted interventions to attract certain subgroups of users that are not satisfied or not reached with the initial intervention. Furthermore, attracting users to the intervention multiple times allows for repeated exposure to the content of the intervention and assimilation of the effective components within the intervention, resulting in an increased likelihood of actual behaviour change. Therefore, focus within the current project is also directed at developing and testing a proactive strategy that may be used to increase sustained use of the intervention over time. Findings from this study are relevant for the current program, but also for other existing and future interventions. Even though a proactive strategy is a relatively simple method and therefore often used in current online health promoting interventions, little is known about their positive or even adverse effects [34]. Therefore, the current study aims at testing the effect of using a proactive approach entailing periodic e-mail prompts compared to a reactive approach on re-visits to an online CT lifestyle program. When periodic prompts are proven to be effective in stimulating sustained use and, possibly, behaviour change and improved health, this could lead to specific recommendations on the use of prompts in online lifestyle interventions. Also, with regard to the use of periodic prompting, results from this study may be further refined by investigating not only the optimal frequency and methods by which respondents should be prompted, but also the content of the prompts.

## List of abbreviations

CT: Computer tailoring; RPHS: Regional Public Health Service; SQUASH: Short QUestionnaire to ASsess Health-enhancing physical activity; QFV: Quantity-Frequency-Variability questionnaire.

## Competing interests

The authors declare that they have no competing interests.

## Authors' contributions

FS significantly contributed to writing this paper, while LvO, DS, MvA, SK and HdV were involved in revising the manuscript. The project design was developed by HdV. All authors were involved in implementing the project. All authors have approved of the version to be published.

## Pre-publication history

The pre-publication history for this paper can be accessed here:

http://www.biomedcentral.com/1471-2458/11/480/prepub
